# Consequences of intra-canopy and top LED lighting for uniformity of light distribution in a tomato crop

**DOI:** 10.3389/fpls.2023.1012529

**Published:** 2023-01-19

**Authors:** R. Schipper, M. van der Meer, P.H.B. de Visser, E. Heuvelink, L.F.M. Marcelis

**Affiliations:** ^1^ Horticulture and Product Physiology, Wageningen University, Wageningen, Netherlands; ^2^ Business Unit Greenhouse Horticulture, Wageningen Research, Wageningen, Netherlands

**Keywords:** functional structural plant model (FSPM), light emitting diode (LED), tomato, interlighting, ray tracing

## Abstract

In the past decade, the potential of positioning LED lamps in between the canopy (intra-canopy) to enhance crop growth and yield has been explored in greenhouse cultivation. Changes in spatial heterogeneity of light absorption that come with the introduction of intra-canopy lighting have not been thoroughly explored. We calibrated and validated an existing functional structural plant model (FSPM), which combines plant morphology with a ray tracing model to estimate light absorption at leaflet level. This FSPM was used to visualize the light environment in a tomato crop illuminated with intra-canopy lighting, top lighting or a combination of both. Model validation of light absorption of individual leaves showed a good fit (R^2^ = 0.93) between measured and modelled light absorption of the canopy. Canopy light distribution was then quantified and visualized in three voxel directions by means of average absorbed photosynthetic photon flux density (PPFD) and coefficient of variation (CV) within that voxel. Simulations showed that the variation coefficient within horizontal direction was higher for intra-canopy lighting than top lighting (CV=48% versus CV= 43%), while the combination of intra-canopy lighting and top lighting yielded the lowest CV (37%). Combined intra-canopy and top lighting (50/50%) had in all directions a more uniform light absorption than intra-canopy or top lighting alone. The variation was minimal when the ratio of PPFD from intra-canopy to top lighting was about 1, and increased when this ratio increased or decreased. Intra-canopy lighting resulted in 8% higher total light absorption than top lighting, while combining 50% intra-canopy lighting with 50% top lighting, increased light absorption by 4%. Variation in light distribution was further reduced when the intra-canopy LEDs were distributed over strings at four instead of two heights. When positioning LED lamps to illuminate a canopy both total light absorption and light distribution have to be considered.

## Introduction

In the past decade, the potential of intra-canopy lighting with light emitting diodes (LED) to enhance crop growth has been explored in greenhouse cultivation, where part of the supplementary light is given from within the canopy. By providing supplementary light within the crop reflective loss of the upper canopy is reduced (Trouwborst et al., 2010). Furthermore, a more homogeneous vertical light distribution and therefore a higher photosynthetic use efficiency of the absorbed light could be achieved (Trouwborst et al., 2010). Studies with intra-canopy lighting partially replacing top lighting showed increased fruit yield in cucumber (Hovi et al., 2004; Hovi-Pekkanen and Tahvonen, 2008), an increase in sweet pepper fruit number and weight (Hovi-Pekkanen et al., 2006), and an increased net photosynthesis (PN) and photosynthetic capacity (Pmax) of cucumber leaves (Pettersen et al., 2010). In other studies no differences were found in whole plant biomass production or yield between top lighting and intra-canopy lighting (Trouwborst et al., 2010; Dueck et al., 2012; Gómez and Mitchell, 2016; Yan et al., 2018). This lack of biomass gain could be related to a loss of total light interception due to extreme leaf curling by intra-canopy lighting as observed by Trouwborst et al. (2010) who used a large fraction of blue light. Gómez and Mitchell, 2016 mentioned an increased maintenance respiration of leaves lower in the canopy that acclimated to a higher light intensity and an increased partitioning to non-harvestable organs as possible reasons for lack of effect on yield. All the explanations mentioned in literature about effects or absence of effects are based on differences in incident light environment which affect plant growth and morphology.

Measuring the incident light environment for individual leaves in a canopy is complex due to large horizontal and vertical light heterogeneity within a canopy. Incident light from lighting from above can be measured by quantum sensors pointing upward at different heights. Incident light from intra-canopy lighting might be measured by use of quantum sensors pointing in a sideward direction towards the intra-canopy lighting at different heights as for instance was done in Kaiser et al. (2019). Total light absorption can be estimated by measuring incident light by a quantum sensors pointing upward and measuring the non-absorbed light by quantum sensors above and below the crop, pointing, respectively, downward and upward. However, the spatial heterogeneity of incident and absorbed light is hard to measure in detail. The spatial variability of absorbed light is important when physiological processes such as photosynthesis are compared between light treatments.

A way to estimate spatial variability in light absorption is through crop modelling. Multiple process based crop models (PBM) have been developed that include the relation between light interception and physiological processes such as photosynthesis. Many crop models use the Lambert-Beer equation (Monsi and Saeki, 2005) to estimate the light interception of a crop, based on the exponential decrease in light intensity with leaf area index (LAI) from top to bottom. With the addition of intra-canopy lighting the vertical light distribution cannot be simply represented by the Lambert-Beer calculation, while it may also have consequences for the horizontal light distribution, which is not considered in models using the Lambert-Beer equation. For simulation of intra-canopy lighting functional-structural plant models (FSPM) might be used. FSPM allow for a more detailed approach which combines 3D plant architecture and a ray tracing model to create understanding of the interaction between plant morphology, light interception, absorbance and distribution patterns at leaf level (Chelle and Andrieu, 2007; Vos et al., 2007; Chenu et al., 2008; Vos et al., 2010; Sarlikioti et al., 2011b; De Visser et al., 2014).

Various FSPM studies have been conducted for greenhouse cultivated crops, such as cut- rose Buck-Sorlin et al., 2011 Zhang et al., 2021) and tomato (Sarlikioti et al., 2011a; Sarlikioti et al., 2011b; De Visser et al., 2010; Kang et al., 2011; De Visser et al., 2014). Those studies aim to either optimize lighting strategies, plant architecture or planting densities to increase interception of light. To our knowledge however, the differences in light heterogeneity when comparing intra-canopy with top lighting have not yet been approached using an FSPM. Uniformity of spatial distribution of light over the leaves is important for maximizing crop photosynthesis. Due to the curvilinear shape of the light response curve of leaf photosynthesis the crop photosynthesis is higher when all leaves have the same intensity, compared to a situation with the same average light intensity but with variation among individual leaves. The importance of uniform light distribution was shown by Li et al. (2014) who found that a more uniform distribution of natural light in the greenhouse by diffuse greenhouse cover (a cover that converted 71% of the direct light into diffuse light) increased crop photosynthesis by 7%.

The objective of this study was to identify the impact of intra-canopy versus top lighting on 3D light distributions in the canopy. This was approached by conducting a greenhouse tomato experiment to parameterize and validate an FSPM which included simulation of light distribution by ray tracing in a 3D environment. This FSPM was then used to simulate and compare light absorption profiles between tomato canopies with intra-canopy lighting, top lighting or a combination of both intra-canopy and top lighting. The heterogeneity of light absorption was approached at the leaflet level in three directions; parallel and perpendicular to the double rows, as well as in the vertical direction.

## Material and methods

### Plant material, growth conditions and light treatments

Tomato plants (*Solanum lycopersicum* L. “Foundation”; Nunhems, Haelen, the Netherlands) were transplanted on the 10*^th^* of February 2017 (52 days after sowing, DAS) in a glasshouse at Wageningen University, the Netherlands (52°N, 5.5°E) and grown until the 1*^st^* of June 2017. The details of the experimental setup can be found in Kaiser et al. (2019), as the data acquired for this study were independently collected during the same experiment. Plants were grown on stone wool slabs (Grodan, Roermond, The Netherlands) for 111 days in a “high wire” system at 2.4 plants m^−2^. Dimensions of the compartment were 6 by 12 meter. The plants were set up in 8 double rows with 1.5 meter double row distance. Each double row of 5m consisted of 20 plants. The glasshouse compartment was kept at 22/16°C day/night temperature, a relative humidity of 78% and 500 ppm CO_2_ partial pressure was applied. This entailed removal of all side shoots, except for the axillary bud just after the sixth truss. All side walls of the greenhouse compartment were covered with a reflective screen, to prevent light pollution from neighboring compartments. In the greenhouse there was a gradient in rows receiving only red LED light to rows receiving up to 24% blue (76% red) light (see Kaiser et al., 2019). In this study rows receiving only red LED were used. Intra-canopy and top red supplemental light was provided by Greenpower PM-DR150 (Philips, Eindhoven, the Netherlands). Led light was supplied as top light (99 μmol m^-2^ s^-1^) and intra-canopy (48 μmol m^-2^ s^-1^) LEDs. The lamps for top lighting were pointing downward, while the lamps for intra-canopy lighting were pointing sideward to the plant rows on both sides. Lamps were on for 16 h per day, unless outside global radiation exceeded 450 W m^−2^. Two LED strings (i.e. fixtures) of intra-canopy lighting were positioned between the plants in the double row at heights of 108 and 153 cm. Plant height during measurement period was 2m.

### Plant architecture measurements

On 20 March (80 DAS), morphological traits of 6 plants were assessed. Stem and internode length, and leaf width and length were measured with a flexible ruler. Number of leaves and leaflets per leaf were counted. Petiole angle, first and second main rachis angles, and the insertion and tip angle of the two biggest leaflets per leaf were measured with a protractor. Leaf length and width, and all angles were measured for rank number 4, 8, 12, 16 and 20, where rank 1 corresponded with the youngest leaf (*>* 2 cm) of the plant.

### Light measurements

**Figure 1:**
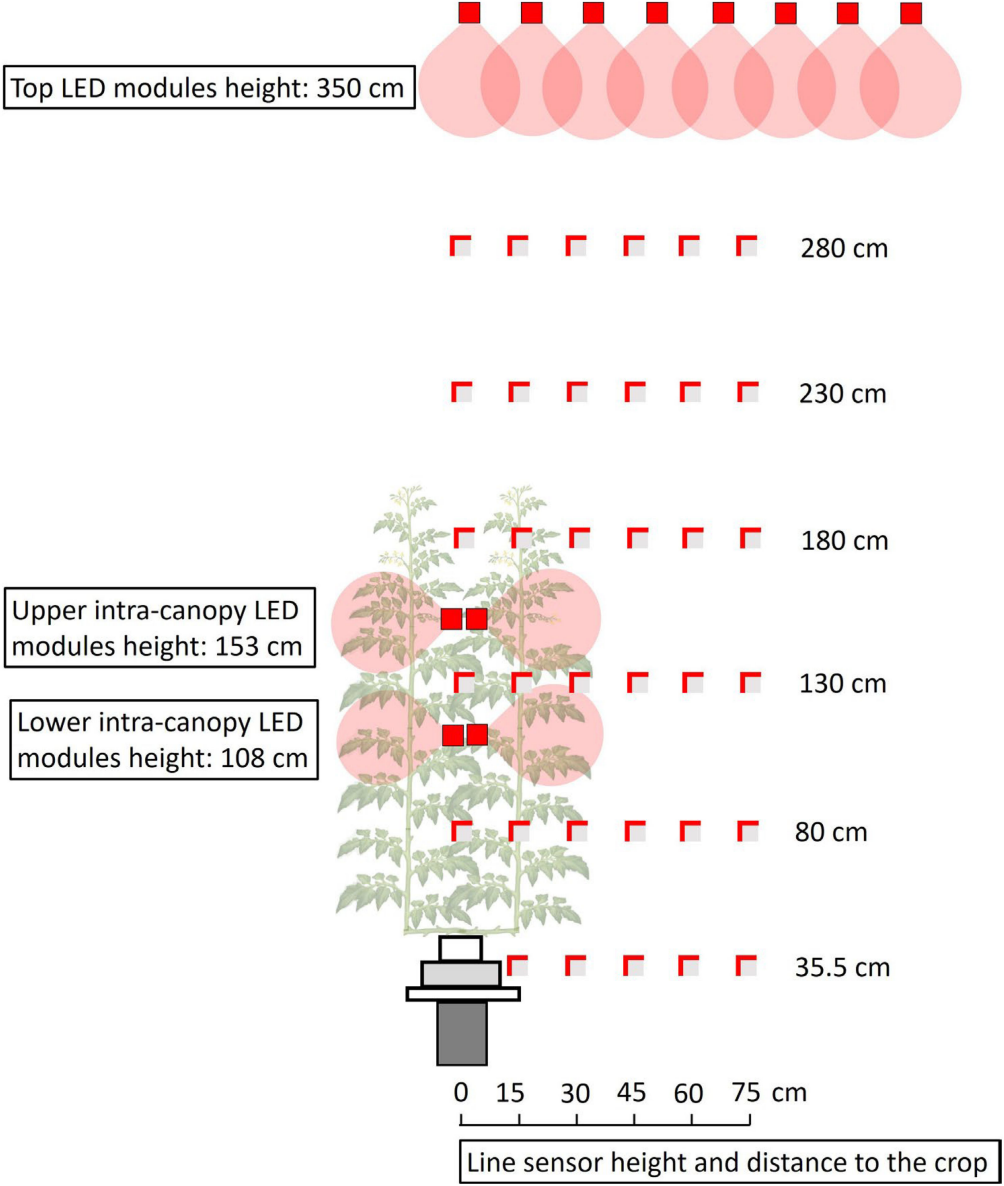
Side view of the light measurements in a tomato crop with LED lamps on top of the canopy and as intra-canopy lighting. Measurements were taken with a line sensor, indicated as a grey square with partly red borders. The line sensor was positioned parallel to the tomato double row at fixed distances (0, 15, 30, 45, 60 or 75 cm) from the center of the double row at fixed heights (35.5, 80, 130, 180, 230 or 280 cm). The two red borders on each grey square indicate the sides to which the line sensor was oriented in order to measure the vertical and horizontal incident light separately. Measurements for intra-canopy and top lighting were done when there was only light from one of the lighting systems at a time.

Vertical and horizontal light distribution was measured 0 to 2 days after the architecture measurements. A grid of photosynthetically active radiation (PAR) measurements was created (**Figure 1**), using one line quantum sensor (1 m, LI-191SA, LI-COR Biosciences, Lincoln, NE, United States) connected to a LI-1400 Datalogger (Li-cor). The sensor was oriented upwards and positioned parallel to the gutter at regular intervals of about 50 cm in the vertical plane (at 35.5, 80, 130, 180, 230 and 280 cm from the floor). To measure the horizontal PAR distribution at each height, the sensor was oriented sideward (towards the intra-canopy lighting) at regular intervals of 15 cm in the horizontal plane (at 0, 15, 30, 45, 60 and 75 cm from the center of the double rows). In addition, reflection from the floor was measured at 41 cm from the floor with the line sensor oriented downwards. All measurements took place in the absence of solar light, at least 2 hours after sunset. Then, either the top lighting, or the upper or lower positioned LED modules were switched on. The same light measurements were done in the greenhouse without plants.

### Model description

An adapted version of a static greenhouse tomato functional structural plant model (De Visser et al., 2014; Van Der Meer et al., 2021) was used. This model was developed on the GroIMP platform (Kniemeyer, 2008) and consists of an architectural and a light module.

#### Plant architecture

The architectural parameter values for leaf length, leaf width, internode length, petiole angle, rachis angles and leaflet angles were taken from phytomer ranks 4, 8, 12, 16 and 20, acquired from the measurements on 20 March (80 DAS). Mean and standard deviation (SD) values for all architectural parameters for the non-measured phytomer ranks were linearly interpolated from the measured phytomer ranks [see Supporting Information-**Figure S1**]. Area per leaf was estimated using a power function fit with leaf width as regressor (leaf area = 0.203*Lw ^1.674^, where *Lw* is leaf width; Schwarz and Kläring, 2001).

**Figure 2:**
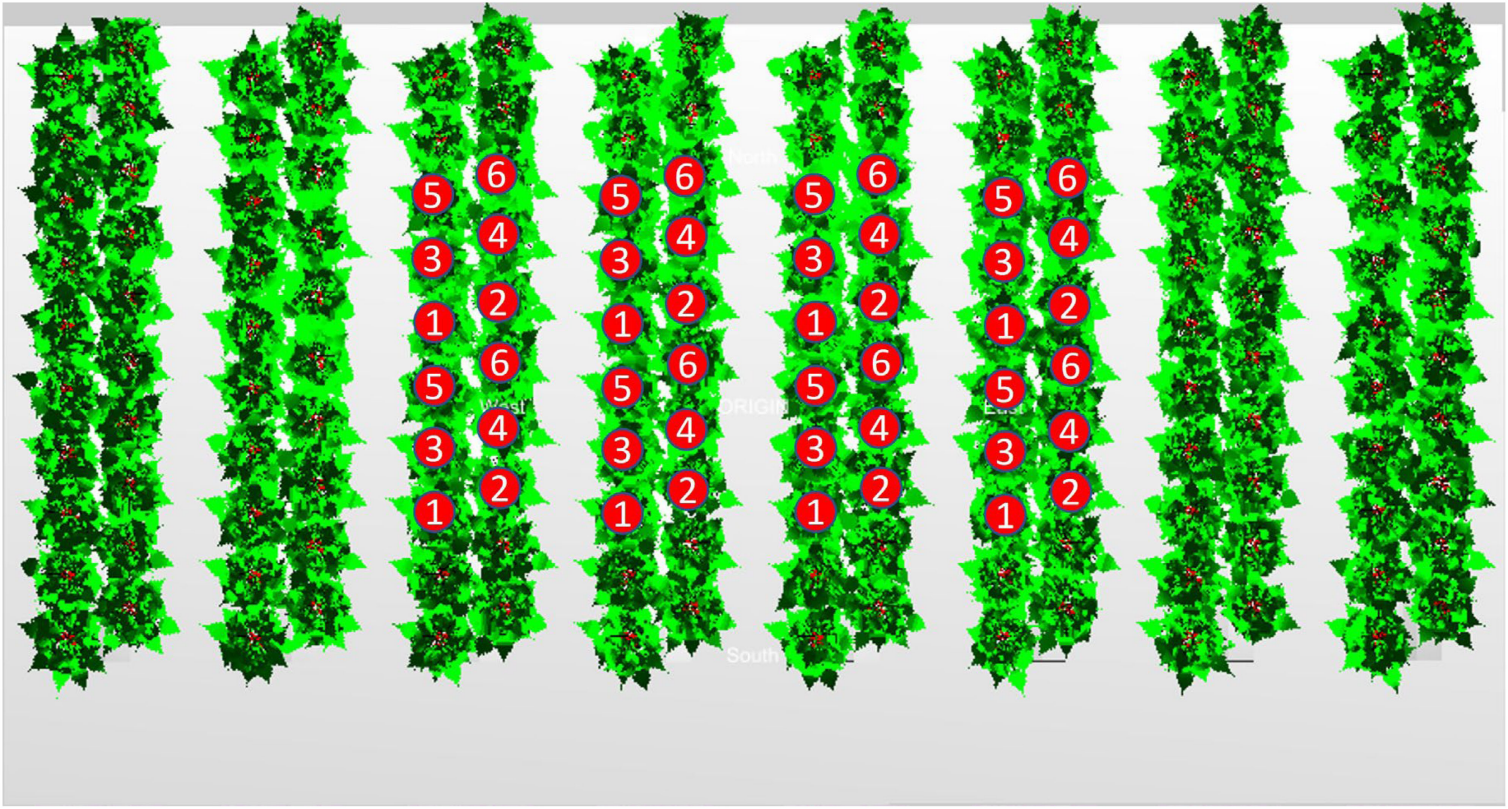
Top view of the reconstructed modelled tomato canopy with architectural parameters measured on 20 March (80 DAS). In each simulation two times six center plants were observed in each of four double rows. These eight groups of six plants each were considered repetitions of each other and the absorbed light of each leaflet was used for further calculations.

Parameter values for the equation were taken from the same paper of Schwarz and Kl¨aring (2001). The leaf area was then distributed across the 11 leaflets (3 bigger pairs, 2 smaller pairs and one terminal leaflet) of each leaf according to an empirical allometric relationships determined by Van Der Meer et al. (2021). Leaflet lengths were then calculated by use of leaf area and leaflet shape according to Evers et al. (2006), after which each leaflet was constructed and represented as 10 parallelograms [see Supporting Information-**Figure S2**]. Modelled architectural parameter values were acquired by drawing values from a normal distribution around the average values measured for each architectural trait. When drawing from a normal distribution, incidentally very small non-realistic values might occur. Therefore, internode length and leaf area were set at a minimum of 0.1 cm and 5 cm^2^, respectively. The apex height of the plants was set at 2 m from the floor, with the intra-canopy lighting on 1.53 and 1.08 m from the floor. The entire canopy comprised all the plants, i.e. 8 double rows of 20 plants each (**Figure 2**). This simulated canopy was defined as the reference canopy.

#### Light module

The LEDs were reconstructed as they were in the experiment using emission patterns according to the lighting company. The light distribution at a given time step was computed by the GroIMP radiation model, which is based on an inversed path tracer with a Monte Carlo pseudo-random number generator as in Veach (1998), which was upgraded to a full-spectral ray tracer by Henke and Buck-Sorlin (2017).

#### Greenhouse environment

The compartment was reconstructed with its major components. The white outside curtains (ILS Hortiroll Revolux w/w) were assumed to have a diffuse reflectivity of 25% for the front and left side wall (since these were behind glass) and 50% for the white curtain on the right wall (since this was inside the compartment). The white plastic that split the treatments in half was assumed to have a diffuse reflectivity of 65%, for the concrete floor this was 30% and for the plastic-covered stone wool slabs 65%. The simulations in the greenhouse were performed for moments that there was no solar radiation, nor was reflection from the greenhouse cover simulated.

#### Model validation

The simulated distribution of LED light inside the greenhouse compartment including plants was compared with the light measurement values performed with the line sensor during the experiment. To quantify the accuracy of the simulated light distribution, in the model virtual line sensors were placed similar to the actual measurements (**Figure 1**). The light intensity at each virtual position of the line sensor was compared with the actual light measurements. The performance of the model was evaluated by the goodness of fit (R^2^).

#### Simulation scenarios

Simulations were run with the reference canopy structure for three different lighting strategies: intra-canopy lighting, top lighting and a combination of intra-canopy and top lighting (50/50%). In each scenario the incident light of all light sources together was equal (85.5 *µ*mol m^−2^ s^−1^), being verified in the light model by enclosing the lamps in a black box absorbing all emitted lamp light. The top lighting modules were placed centered above each double row. Intra-canopy lighting modules were simulated at 108 and 153 cm height similar as in the experimental setup.

#### Evaluation of simulated light distribution and heterogeneity

In each simulation two times six center plants were observed in each of four double rows (**Figure 2**). These eight groups of six plants each were considered repetitions of each other for further calculations. Each of these sets of six simulated plants was divided into voxels to visualize the canopy light distribution. Each voxel had a width and height of 7.5 cm and a length to include all leaflets of the considered plants within that direction. The voxels were directed either (1) horizontal parallel to the row, (2) horizontal perpendicular to the row or (3) vertical. Consequently, the corresponding length of the voxels were based on either (1) the row length of the six plants in the row, (2) the width of four double plant rows (hence includes 8 plants) or (3) the height of the plants.

**Figure 3:**
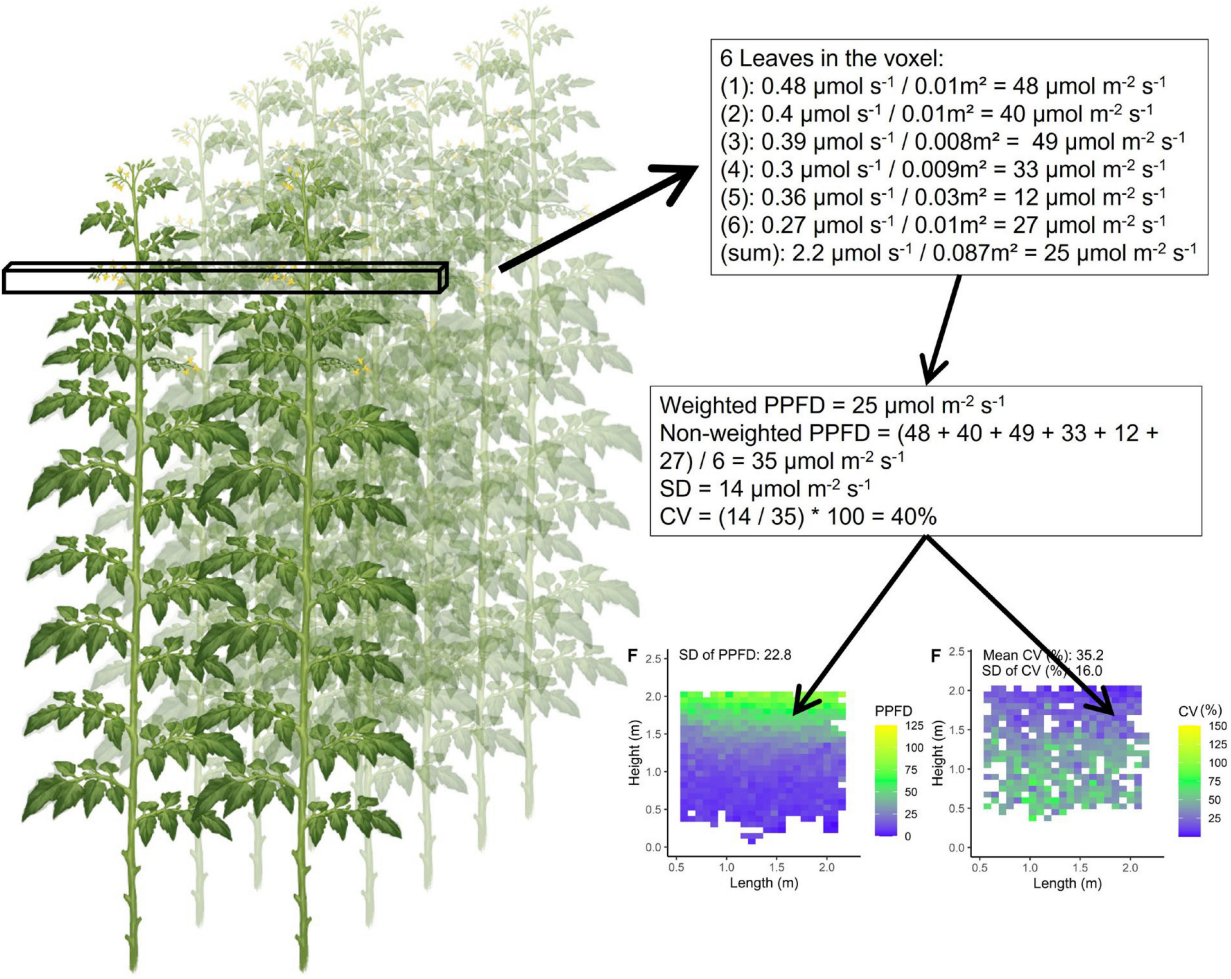
Calculation of the coefficient of variation (CV) and mean leaflet absorbed PPFD within one voxel (perpendicular to the plant row) of the tomato plant canopy. Each voxel had the dimensions of 7.5 cm width and length and a depth reaching across four double rows (8 plants). As a hypothetical example calculated values are shown. Subsequently two figures are produced to visualise the PPFD of each voxel (voxels perpendicular to the rows) in the canopy and the coefficient of variation within each voxel.

For each voxel, the average absorbed PPFD (*µ*mol m^−2^ s^−1^) and coefficient of variation (CV) within that voxel were calculated. This was done with light absorption data collected for each individual leaflet (*µ*mol s^−1^ per leaflet; for sample sizes in each voxel see Supporting Information-**Figure S3**). The average absorbed PPFD inside each voxel was calculated by dividing the cumulative absorbed light of all leaflets by the cumulative leaf area (m^2^) of all leaflets inside the voxel (**Figure 3**). Furthermore, the average, SD and CV of absorbed PPFD was calculated based on average values of each voxel. These SD and CV values give an indication of heterogeneity between voxels, quantifying the heterogeneity within the canopy. For calculating the CV within a voxel the four smallest leaflets on each leaf were not considered due to their small total fraction (0.09%) of the leaf area. By excluding these four smallest leaflets the calculated CV is representative for the majority of the photosynthetically active leaf area. Then, inside each voxel the absorbed PPFD was calculated for each leaflet individually and these were then used to calculate the mean absorbed PPFD with its associated standard deviation (SD) between leaflets. The CV was then calculated by dividing the SD by the mean absorbed PPFD. Furthermore, a mean CV of canopy absorbed PPFD was calculated by averaging the CV of each voxel (indicating the average CV within a canopy).

#### Sensitivity analysis

Besides the reference simulations there were additional model simulations that tested the sensitivity of the mean CV and SD of the absorbed PPFD to changes in height and number of the LED modules and the ratio between intra-canopy and top light. Either the height of the LED modules were increased or decreased by 30 cm, the number of LED module strings was increased from 2 to 4 (the additional 2 LED module strings were located at 131 and 86 cm from the floor, while the original two LED module strings were at 108 and 153cm height; see Supporting Information-**Figure S4**), or the ratio between intra-canopy and top light was set at 25/75% or 75/25%.

## Results

### Validation of the model

**Figure 4:**
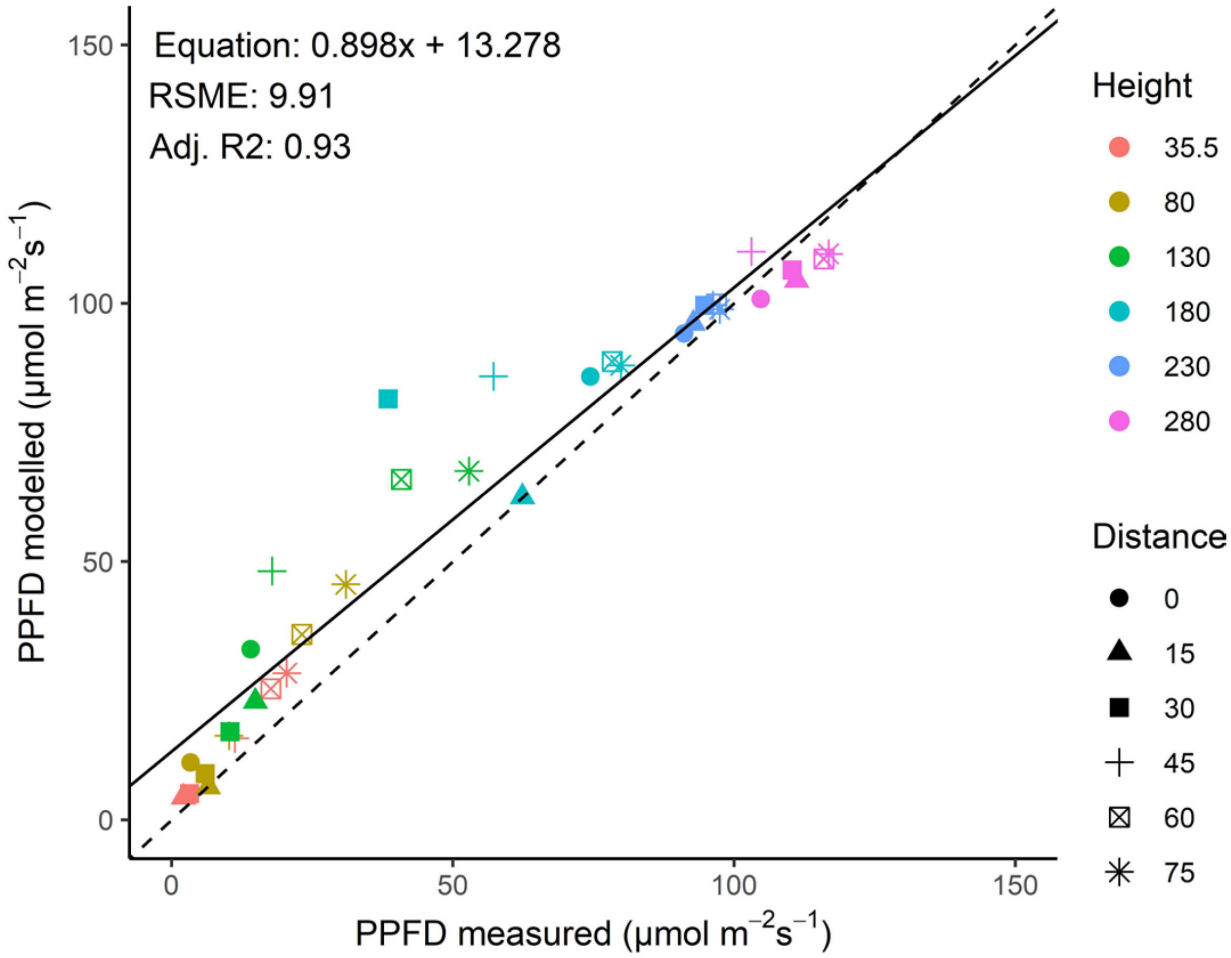
Relationship between measured and modelled incident PPFD (as observed by upward-facing sensors at different heights) in a greenhouse with a tomato crop illuminated by top lighting. The different symbols represent different distances to the middle of the rows in the canopy, whereas the symbol color represents height from the floor. Position of plants, lamps and sensors is shown in **Figure 1**.

Measured and modelled incident PPFD (as observed by upward-facing sensors at different heights) showed a good correlation (R^2^ = 0.93; **Figure 4**). A sensitivity analysis showed that an increased leaf length of +25%, an increase in stem width of +50% or an increase of apex height by +30 cm had slight positive effects on the goodness of fit by up to 4% [see Supporting Information-**Figure S5**]. Therefore, since all of the fits were with a high R^2^ no modifications were made based on the sensitivity analysis.

#### Light distribution and heterogeneity with top lighting and intra-canopy lighting

**Figure 5:**
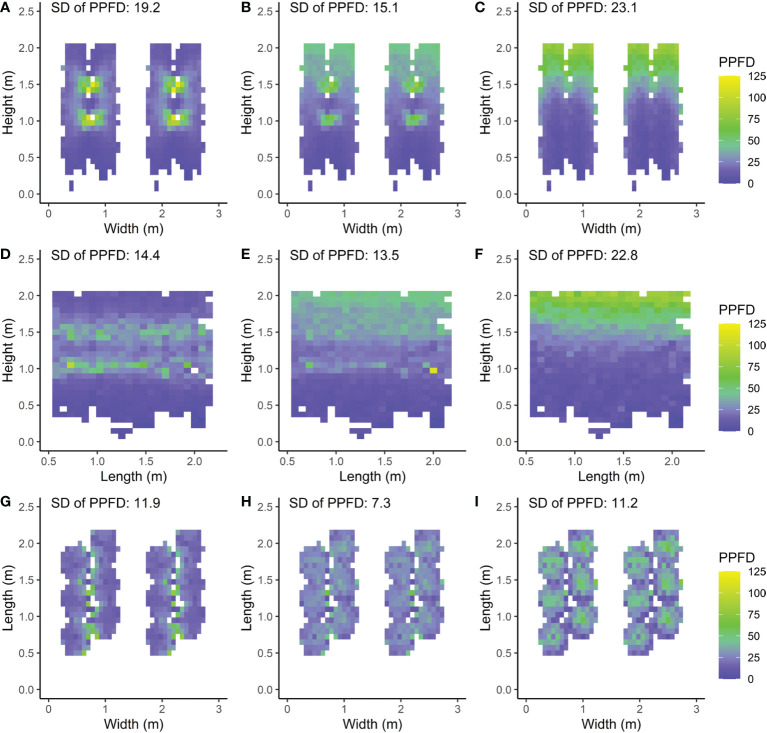
Front **(A–C)**, side **(D–F)** and top views **(G–I)** of a double row canopy with mean absorbed PPFD (*µ*mol m*^−^* ^2^ s*^−^* ^1^) per voxel calculated as the mean taken from each leaflet’s absorbed PPFD within the voxel. Three LED lamp positions are compared; **(A, D, G)** intra-canopy lighting, **(B, E, H)** combined intra-canopy and top lighting, and **(C, F, I)** top lighting. Each voxel had the dimensions of 7.5 cm width and length and a depth reaching 6 plants in each row **(A–C)**, 8 plants across four double rows **(D–F)** or whole plant height **(G–I)**. This means that the voxels are directed **(A–C)** parallel to the row; **(D–F)** perpendicular to the row and **(G–I)** vertically. The standard deviation (SD) is calculated over all voxels. Plants are spaced at 0.5m within each row in a double row. For visual interpretation of the distance between rows in the canopy an additional replicate double row is shown in **A–C**, **G–I** for which the same values were used as in the other row.

**Figure 6:**
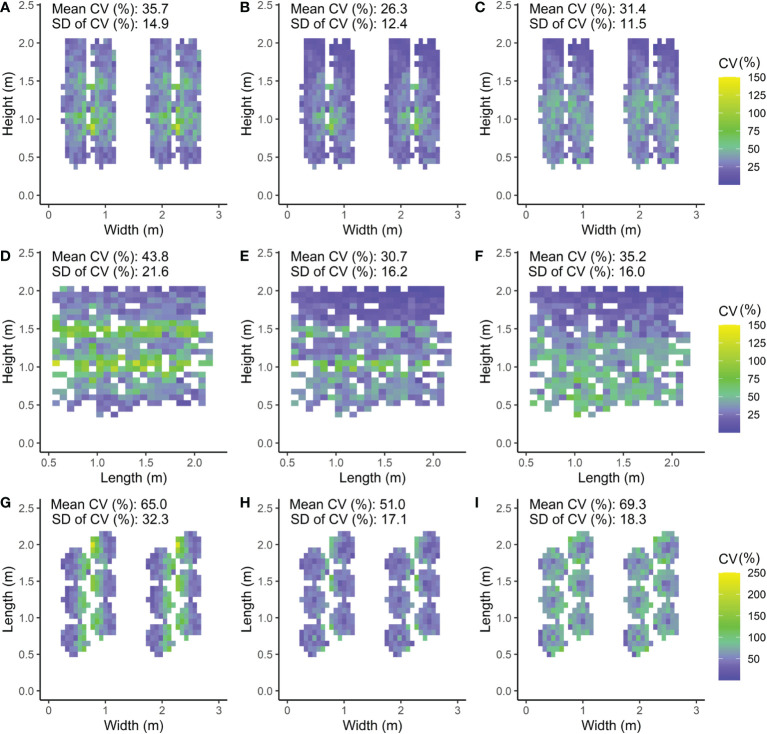
Front **(A–C)**, side **(D–F)** and top views **(G,H,J)** of the canopy with the CV (coefficient of variation) calculated as the standard deviation (SD) of the absorbed PPFD divided by the mean absorbed PPFD of the leaflets within the voxel times 100%. Three LED lamp positions are compared; **(A, D, G)** intra-canopy lighting, **(B, E, H)** combined intra-canopy and top lighting, and **(C, F, I)** top lighting. Each voxel had the dimensions of 7.5 cm width and length and a depth reaching 6 plants in each row **(A–C)**, 8 plants across four double rows **(D–F)** or whole plant height **(G–I)**. In A–C, a front view of the canopy is shown in which the CV value represents the heterogeneity of the voxel oriented parallel to the row. In D–F, a side view of the canopy is shown in which the CV value represents the heterogeneity of the voxel oriented perpendicular to the row. In G–I, a top view of the canopy is shown in which the CV value represents the heterogeneity of the voxel oriented vertical. Plants are spaced at 0.5m within each row in a double row. For visual interpretation of the distance between rows in the canopy an additional replicate double row is shown in **A–C**, **G–I** for which the same values were used as in the other row.

The modelled light distribution for the intra-canopy lighting, top lighting and combined lighting system was visualized by 2D heat maps of absorbed PPFD per voxel (**Figure 5**). Additionally, the heterogeneity was expressed as the coefficient of variation within each voxel, visualised by 2D heat maps as well (**Figure 6**).

##### Vertical light distribution averaged parallel to row

The simulated PPFD absorbed by the leaflets was largest for leaflets close to the intra-canopy lighting modules (**Figures 5A–C**). The absorbed PPFD values decreased with distance in a circular distribution pattern from the intra-canopy lighting modules and approached low values even within the same double row in which the intra-canopy lighting modules were located. In particular, the difference was notable in absorbed PPFD on the outsides of the double row compared to the inward row side. When top lighting was added to the intra-canopy lighting while total incident light intensity remained equal, the absorbed PPFD values showed a more homogeneous light distribution pattern through the canopy (**Figure 5B**). For top lighting only, the absorbed PPFD distribution pattern in the vertical plane was much steeper with a larger SD (23.1) compared to either intra-canopy setting (SD of 19.2) or a combined lighting from top and intra-canopy lighting (SD of 15.1; **Figures 5A–C**). Both the horizontal variability perpendicular to the row occurring with sole intra-canopy lighting, and the vertical variability occurring with sole top lighting, were diminished by the combination of intra-canopy and top lighting.

##### Vertical light distribution averaged perpendicular to row

The side view of the canopy showed the light distribution in the vertical plane and parallel to the row (**Figures 5D–F**). With sole intra-canopy lighting (**Figure 5D**), variation throughout the canopy occurred mostly in the vertical direction, when looking at the side view. Interestingly, on the height where the intra-canopy lighting was located, there were quite big differences in absorbed PPFD parallel to the row as well. With a combination of intra-canopy and top lighting the variation in absorbed PPFD slightly decreased when compared to intra-canopy lighting. For top lighting the distribution of absorbed PPFD was more homogeneous across the length of the row, but much less homogeneous with height of the canopy, resulting in larger variation of mean absorbed PPFD compared to intra-canopy lighting or a combination of intra-canopy and top lighting, when looking at the side view (SD being 22.8 versus respectively 14.4 and 13.5; **Figures 5D–F**).

##### Horizontal light distribution averaged over heights

In the horizontal direction absorbed light distribution was more uniform for combined intra-canopy and top lighting (SD of 7.3) compared to intra-canopy lighting (SD of 11.9) or top lighting (SD of 11.3). In general, the horizontal light distribution (averaged over all heights) seemed more homogeneous than the vertical light distribution (averaged over horizontal layers) since (all) SD values were lower when averaging **Figures 5A–I**).

##### Variation coefficient horizontally in length direction of row

The CV of absorbed light within horizontal voxels (7.5 x 7.5 cm x 6 plants) parallel to the rows was smallest for the combined intra-canopy and top lighting; CV of 26%, compared to 36% for intra-canopy lighting and 0.31 for top lighting (**Figures 6A–C**). Consequently, the light uniformity parallel to the row was best for a combined intra-canopy and top lighting setting (**Figure 6B**).

##### Variation coefficient horizontally perpendicular to row

Within horizontal voxels (7.5 x 7.5 cm x 4 double rows) perpendicular to the rows, combined intra-canopy and top lighting showed the lowest mean CV (31%) of absorbed light, followed by top lighting (35%) and then intra-canopy lighting (44%) (**Figures 6D–F**). Therefore, uniformity of absorbed PPFD is lowest for intra-canopy lighting when uniformity perpendicular to the row is considered (**Figure 6D**), and is reduced by combined intra-canopy and top lighting (**Figure 6F**).

##### Variation coefficient vertically

Within vertical voxels (7.5 x 7.5 cm x plant height), combined intra-canopy and top lighting showed the lowest mean CV (51%) of absorbed light, followed by intra-canopy lighting (65%) and top lighting (69%; **Figures 6G–I**). Light heterogeneity is highest for top lighting in the vertical direction of the canopy, and is strongly reduced by combined intra-canopy and top lighting.

##### Average distribution in absorbed PPFD in the vertical and horizontal plane

**Figure 7:**
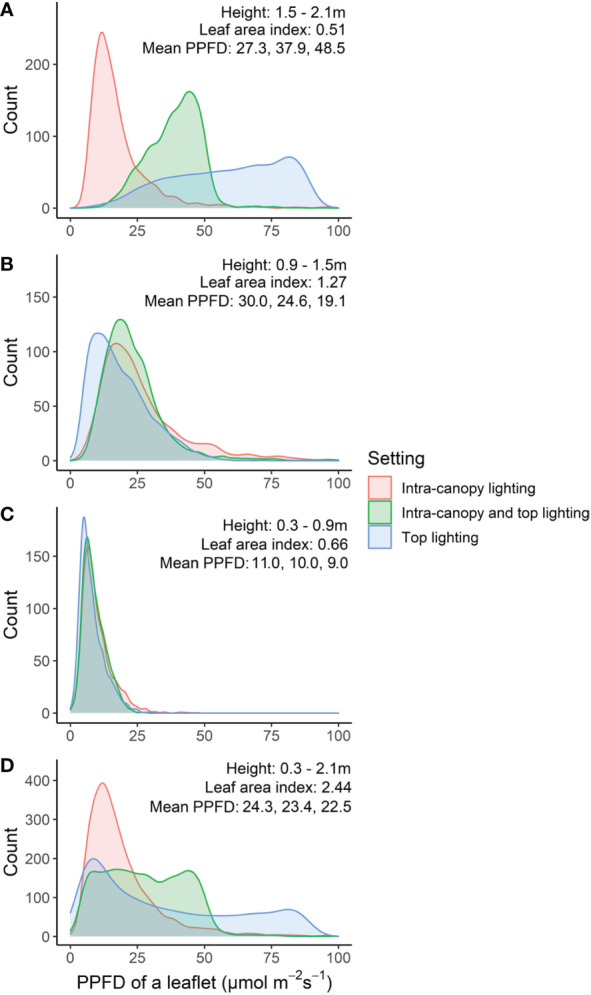
Frequency distribution of leaflets based on absorbed PPFD (*µ*mol m*^−^* ^2^ s*^−^* ^1^) at top **(A)**, middle **(B)** bottom **(C)** or whole of canopy **(D)**. Numbers in each figure indicate the height range, the leaf area index, and the mean weighted absorbed PPFD (*µ*mol m*^−^* ^2^ s*^−^* ^1^) of all leaflets for intra-canopy lighting, combination of intra-canopy and top lighting, and top lighting, respectively. From within the canopy 48 center plants were taken from four center double rows, 6 plants from each double row side.

**Figure 8:**
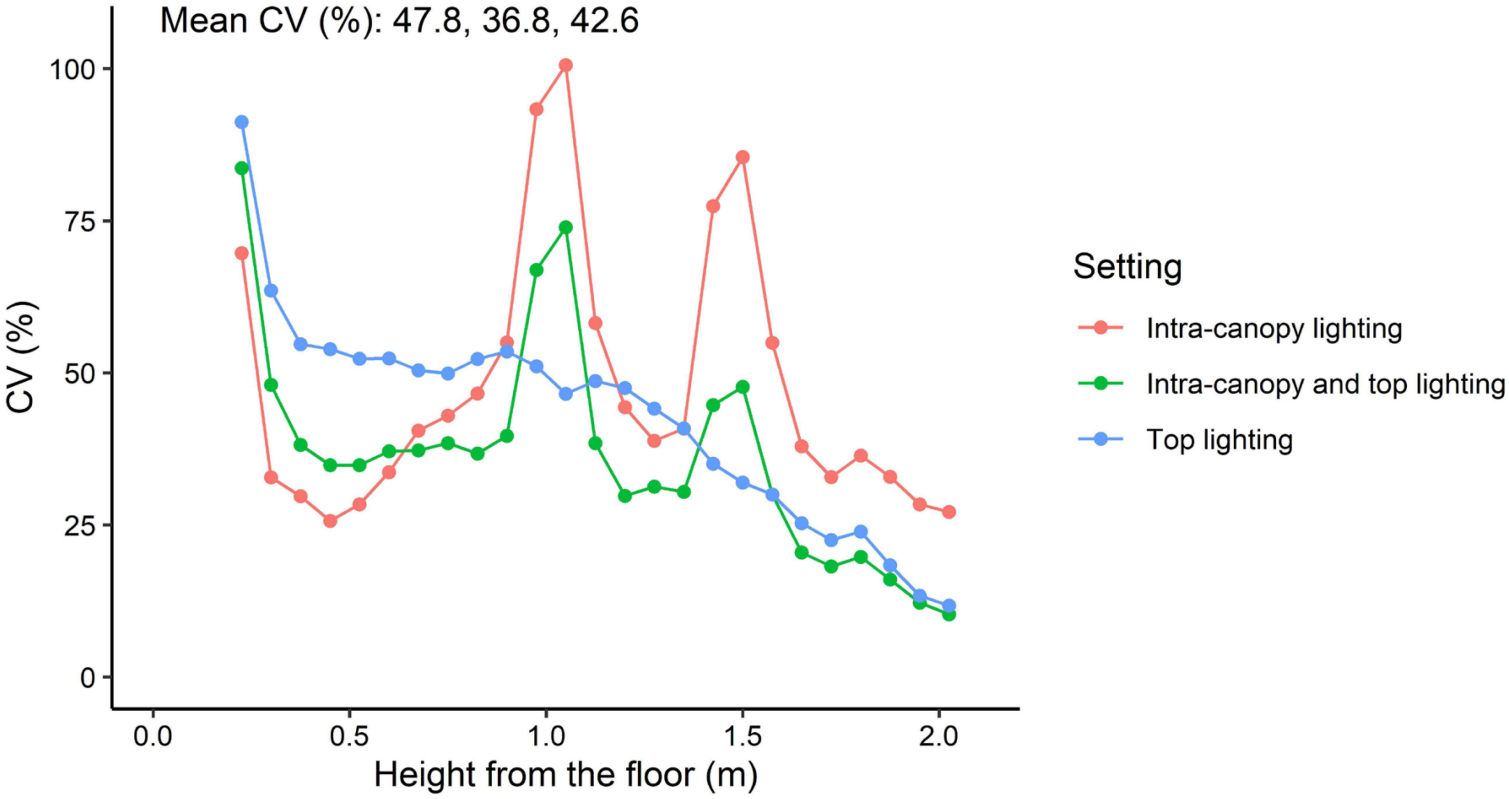
Coefficient of variation (CV) of absorbed PPFD in relation to height in the canopy. CV relates to the variation within horizontal layers. Each layer is represented by 7.5 cm height and takes all perpendicular and parallel leaves of 48 plants and is shown as a dot in the graph. Mean CV of all horizontal layers is provided in the top left corner for intra-canopy lighting; intra-canopy and top lighting; and top lighting, respectively.

Differences between top lighting and intra-canopy lighting occurred at the top and middle of the canopy (**Figures 7A, B**). In the top of the canopy intra-canopy lighting resulted in the lowest weighted absorbed PPFD (27.3 *µ*mol m^−2^ s^−1^ versus 48.5 for top lighting). These differences were the opposite for the middle of the canopy, where the weighted absorbed PPFD was 30.0 *µ*mol m^−2^ s^−1^ for intra-canopy lighting versus 19.1 for top lighting. In the lower part of the canopy the differences were minor, with 11.0 *µ*mol m^−2^ s^−1^ for intra-canopy lighting versus 9.0 for top lighting (**Figure 7C**). The combination of intra-canopy and top lighting was always in between the other two treatments. The mean absorbed PPFD across all leaves in the canopy was highest for intra-canopy lighting, with a value of 24.3 *µ*mol m^−2^ s^−1^, compared to 23.4 for the combination of intra-canopy and top lighting and 22.5 for top lighting only. This indicates that total light absorption was 8% higher for intra-canopy lighting, and 4% for combination of intra-canopy light and top lighting compared than top lighting. The intra-canopy lighting has very high peak values for CV around the height of the LED strings (**Figure 8**). The mean CV and SD over the different horizontal layers demonstrated highest values for intra-canopy lighting (48% *±* 22), followed by top lighting (0.43% *±* 18) and then the combination of intra-canopy and top lighting (37% *±* 18). This means that the variation within the horizontal layers is highest for intra-canopy lighting. The values for radiation mentioned here are related to LED lighting with 85.5 µmol m^−2^ s^−1^ LED light. This leads to relatively low intensities at leaf level, which would proportionally increase when the higher LED light intensity would have been used.

##### Sensitivity analysis of fraction and position of intra-canopy light

**Figure 9:**
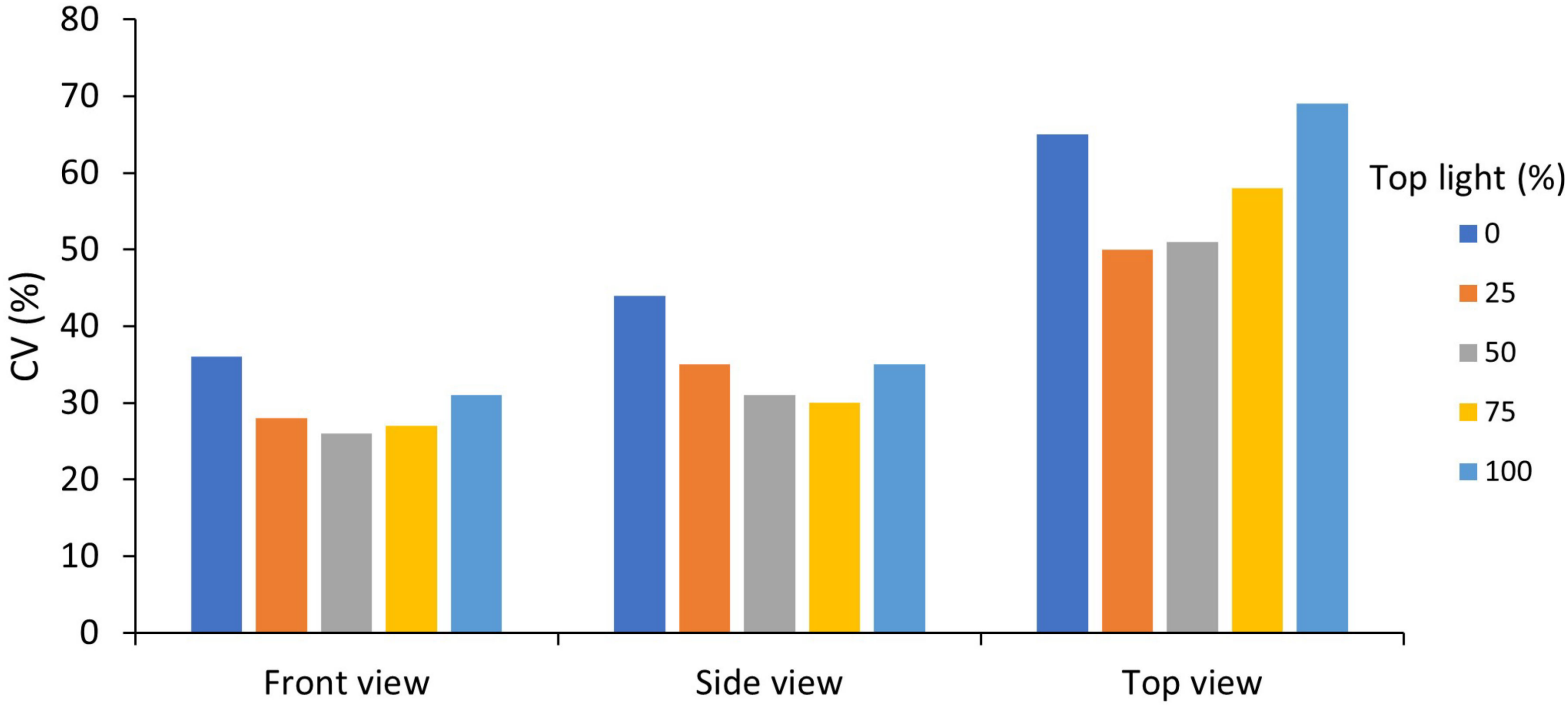
Effects of percentage top light (0, 25, 50, 75 or 100%) on the mean coefficient of variation (CV) within voxels of absorbed canopy PPFD for the front, side and top view of the canopy. Top light percentage was 0, 25, 50, 75 and 100%, with respectively, 100, 75, 50, 25 and 0% intra-canopy light. Each voxel had the dimensions of 7.5 cm width and length and a depth reaching 6 plants in each row of a double row stretching across four double rows.

Changing the ratio between intra-canopy and top lighting showed that 50/50% gave the lowest CV (**Figure 9**). The higher the percentage of either intra-canopy or top light, the higher the CV. Increasing or decreasing the height of the intra-canopy lighting LEDs by 30 cm largely had no effect on the variation in the horizontal plane, but variation in the vertical plane was reduced when height of the intra-canopy light was reduced by 30cm, while it increased when LED strings were raised by 30cm (**Supplementary Figure S6**). Increasing the number of intra-canopy LED strings from two to four strings keeping the same total intra-canopy light output also reduced the CV [see Supporting Information-**Figure S6**].

## Discussion

### Partial replacement of top by intra-canopy lighting increases absorbed PPFD uniformity

In our study we showed that FSPM appears to be an effective tool to visualize and quantify the distinctive extinction patterns throughout the canopy with intra-canopy lighting and/or top lighting. For intra-canopy lighting there is a strong absorption close to the LED modules and the majority of the light does not even reach the outside of the double row in which the intra-canopy lighting is located (**Figure 5A**), which is a similar observation to that of De Visser et al. (2014). This may lead to local acclimation to the areas experiencing high light conditions (Joshi et al., 2019). Despite high local light intensities surrounding intra-canopy LED modules there is a lower overall variation (smaller SD) in absorbed light when compared to top lighting (**Figure 5**). The combination of a higher uniformity (**Figure 5**) and a higher total light absorption (**Figure 7**) all favor intra-canopy lighting above top lighting. It should be realised that younger leaves are generally most photosynthetically active and are acclimated to high light conditions (e.g. Qian et al., 2012). Therefore, a more homogeneous vertical distribution does not necessarily lead to a higher photosynthesis in all cases, as a relative larger fraction of light will be absorbed by leaves with photosynthetic parameters which are less favourable for high rates of photosynthesis. On the other hand when the light profile in the canopy changes, the leaves will acclimate to the changed light profile. Consequently with intra-canopy light the lower leaves will acclimate to relatively higher light levels and therefore be more photosynthetic active and less rapidly senesce (Trouwborst et al., 2011) Our model simulations show that a combination of top lighting with intra-canopy lighting results in the most uniform light distribution in the canopy, when compared to sole intra-canopy as well as compared to sole top lighting. In addition, there is a slight increase in light absorption by 4% compared to top lighting but 4% less compared to sole top lighting. The lower light absorption of top lighting versus intra-canopy lighting, is due to some reflection of top lighting by the top layer of the canopy. So, overall a combination of top and intra-canopy lighting increases uniformity of light distribution over individual leaves and total light absorption of the canopy compared to sole top lighting. op light

Variation in light intensity along the LED string (parallel to the row) was still present despite the string having a LED at every few cm showing the effect of plant-caused variation due to irregularly oriented leaves and randomly occurring open spaces in the crop. It is possible that in reality leaves turn towards the light (as shown for cucumber by Kahlen et al., 2008) and thus reduce their irregular positioning. Research on diffusing solar light has shown that a more uniform light distribution over the laves in the canopy can increase crop photosynthesis and growth Li et al., 2014 Earlier we (Van Der Meer et al., 2021) showed that increased variation in light distribution due to different sun exposures between plant rows hardly resulted in yield reduction in a similar tomato crop, since leaves adapted in size and thickness. Moreover, effects of higher spatial light intensity variation may depend on average light intensity, being larger when light intensities are high as the relative response of leaf photosynthesis slows down at high light intensity. The present calculations were done for red light which is highly absorbed by leaves. If the light would contain a high fraction of green light, the scattering would be larger resulting in a deeper light penetration and slightly more uniform light distribution. Consequences of changed light distribution on morphological and physiological acclimation of leaves and consequence for photosynthesis and growth request for further research.

### Impact on crop production

A combination of intra-canopy and top lighting is recommended for reducing canopy heterogeneity in absorbed light (**Figures 5**, **6**, **8**, **9**). The simulation results also show that there is an optimum for the fraction of intra-canopy light for optimizing uniformity. This optimum was at about 50% intra-canopy light and 50% top light. When there is also solar light (coming from the above) the optimum ratio of intra-canopy lighting to top lighting is likely to increase; most likely the optimum fraction of intra-canopy light is when the intra-canopy light is similar as the total light from top light and sun. Furthermore, the position of the intra-canopy lamps is important. Too high positioning may lead to light loss to the greenhouse cover and too low positioning may lead to light loss to the floor. Distributing the intra-canopy light over different heights in the canopy also increased the light uniformity [see Supporting Information-**Figure S6**].

Experimental comparison between lighting strategies by means of photosynthetic characteristics is difficult for various reasons. The most obvious is that measurements are time consuming and limited in number. Our findings demonstrate that there is a large variation in local light conditions when comparing lighting treatments. Joshi et al. (2019) found a 3-4 times higher photosynthetic capacity for bell pepper at intra-canopy lighting on the inside of the canopy close to the LEDs compared to the control without supplementary lighting. Such acclimation of photosynthesis to changed light intensity may take place within a week (Hogewoning et al., 2007). Apart from acclimation, intra-canopy lighting simulations showed an increased light absorption compared to top lighting. Furthermore, the location of the lamps in the canopy may potentially affect development of individual plant organs and dry matter partitioning and, hence, affect yield

Further experimental studies are needed to investigate effects on light distribution, photosynthesis, growth, and yield by intra-canopy lighting. Studies with intra-canopy lighting partially replacing top lighting showed increased fruit yield in several studies (Hovi et al., 2004; Hovi-Pekkanen et al., 2006; Hovi-Pekkanen and Tahvonen, 2008) but having not effects in other studies (Trouwborst et al., 2010; Dueck et al., 2012; Gómez and Mitchell, 2016; Yan et al., 2018).

## Conclusions

Positioning of LED lamps above or in between the canopy (intra-canopy) has large effects on total canopy light absorption and the distribution of the absorbed light over the leaves. Intra-canopy lighting resulted in 8% higher total light absorption than top lighting, while combining 50% intra-canopy lighting with 50% top lighting, increased light absorption by 4%. Combining intra-canopy and top lighting resulted in a more uniform canopy light absorption than intra-canopy or top lighting alone.

## Data availability statement

The raw data supporting the conclusions of this article will be made available by the authors, without undue reservation.

## Author contributions

MvdM and RS contributed to this chapter equally. MvdM and RS devised the main conceptual ideas. RS designed the measurement protocol and executed architectural as well as light measurements in the experiment. MvdM adapted the functional structural plant model and performed all model simulations. All authors were involved in data analysis. MvdM and RS drafted the manuscript and designed the figures. LM, EH, PdV initiated and supervised the research, and they edited the manuscript.
