# Supplementary material for: Consequences of intra-canopy and top LED lighting for uniformity of light distribution in a tomato crop
Source: Front Plant Sci. 2023 Jan 19;14:1012529. doi: 10.3389/fpls.2023.1012529 (PMC9893118; doi:10.3389/fpls.2023.1012529)
Supplement: Supplementary file 1 [file DataSheet_1.docx]

**Consequences of intra-canopy and top LED lighting for uniformity of light distribution in a tomato crop**

Schipper, R. ^1∗^, van der Meer, M. ^1∗^, de Visser, P.H.B. ^2^, Heuvelink, E. ^1^, Marcelis, L.F.M. ^1#^

# SUPPLEMENTARY MATERIAL


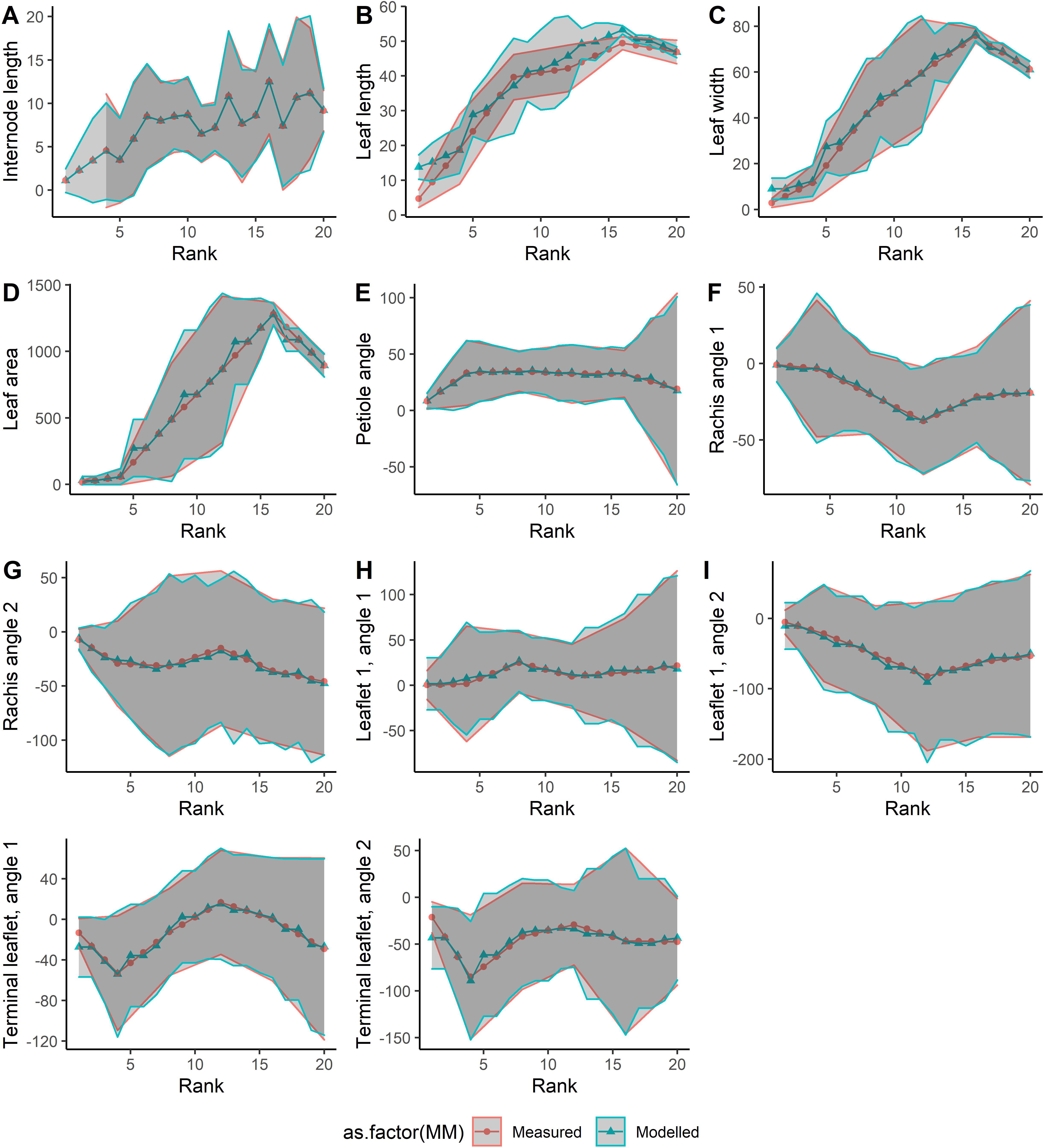


Figure S1: Measured vs modelled mean plant architecture values with confidence interval (2 times standard deviation). The architectural parameter values for leaf length, leaf width, internode length, petiole angle, rachis angles and leaflet angles were taken from phytomer ranks 4, 8, 12, 16 and 20 (counted from the top; from the first leaf 2 cm), acquired from the measurements on 20 March (81 DAT). Mean and standard deviation (SD) values for all architectural parameters of the non-measured phytomer ranks were linearly interpolated between the measured phytomer ranks.


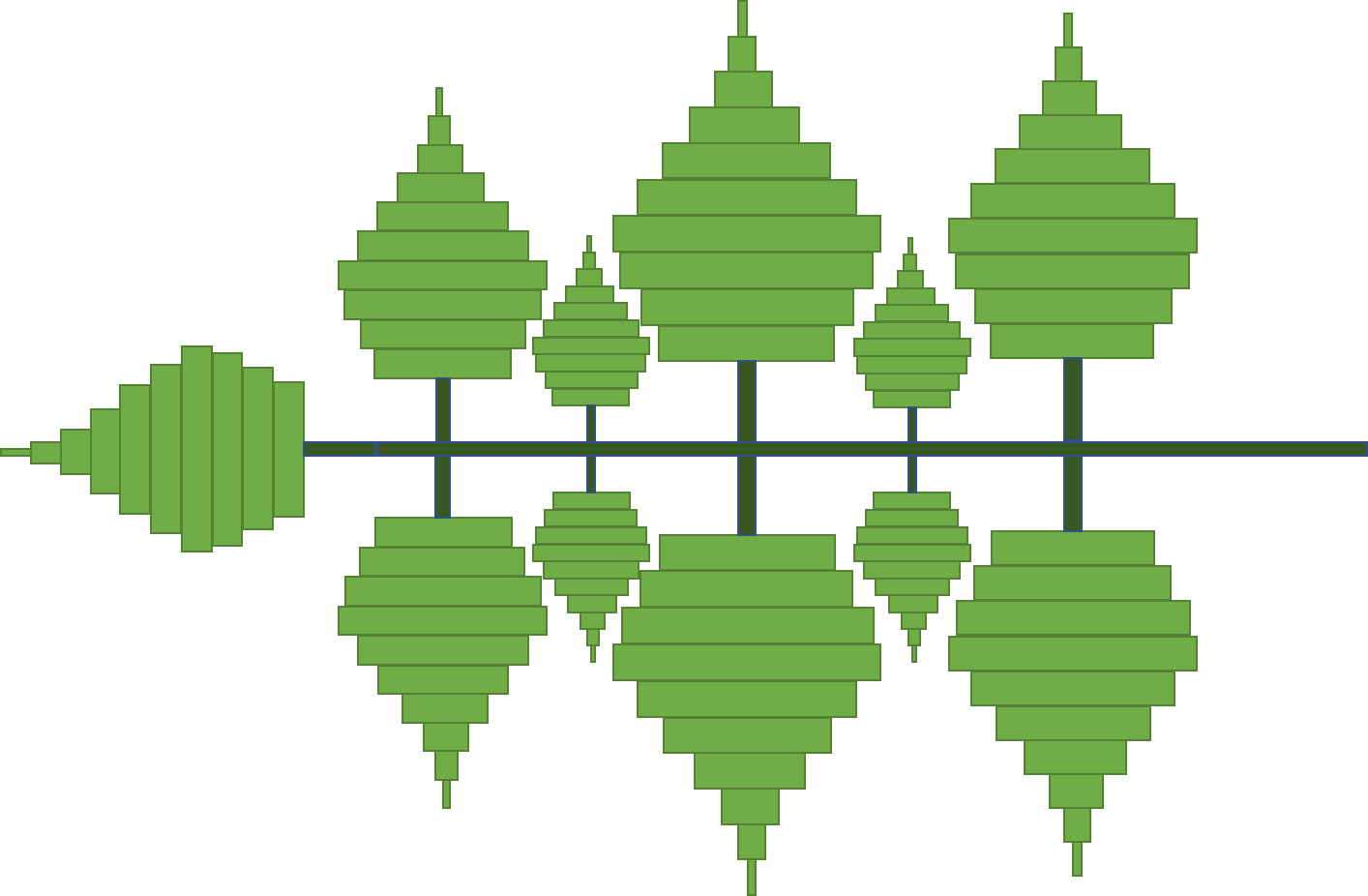


Figure S2: Representation the reconstruction of a single leaf with leaflets as used in the model. Each leaf consisted of 4 small and 7 larger leaflets.


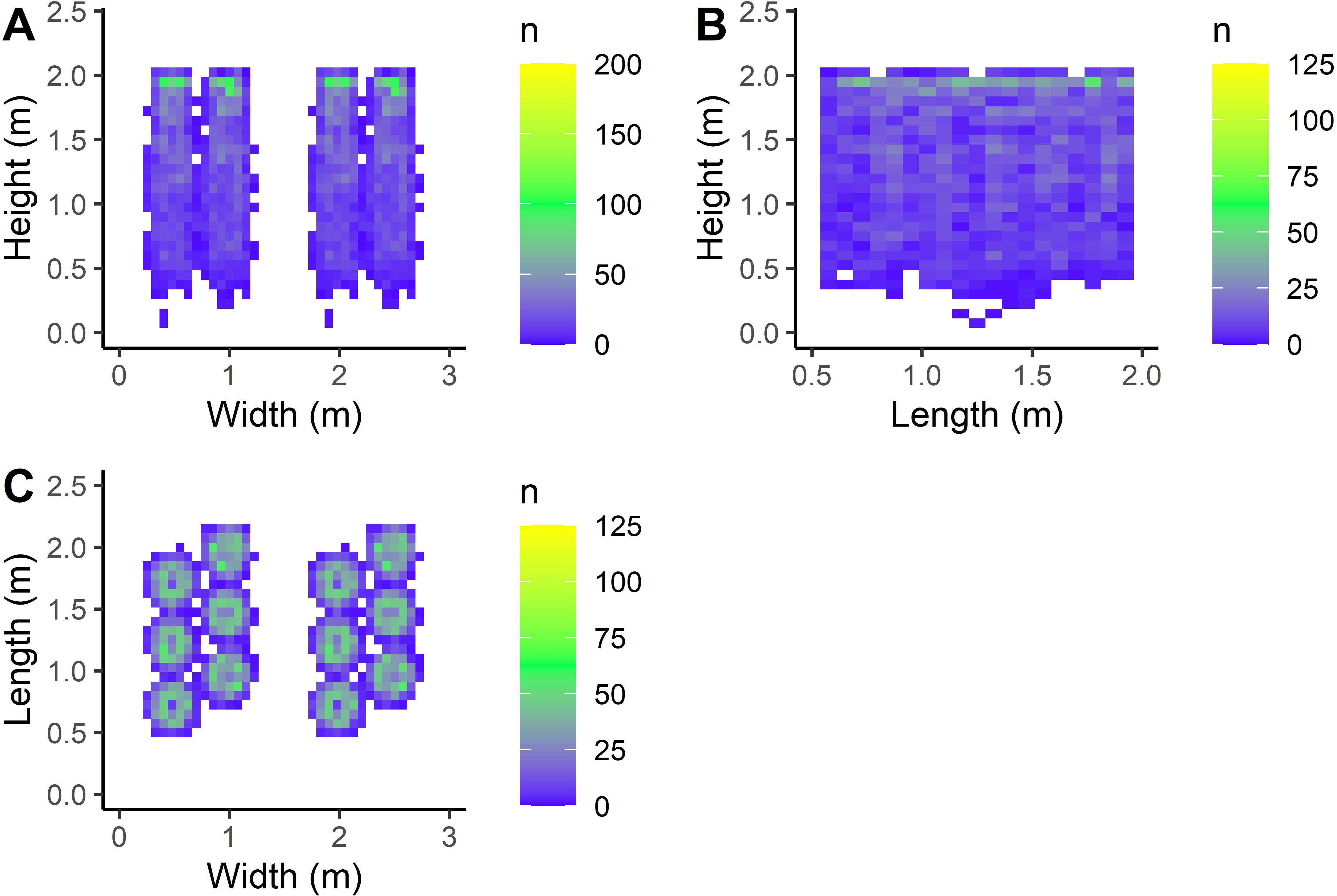


Figure S3: A front, side and top view of a double row canopy with the number of leaflets used for absorbed light calculations within each voxel. Each voxel had the dimensions of 7.5 cm width and length and a depth reaching 6 plants in each row (A), 8 plants across four double rows (B) or whole plant height (C). This means that the voxels are directed (A) parallel to the row; (B) perpendicular to the row and (C) vertically. Plants are spaced at 0.5m within each row in a double row. For visual interpretation of the distance between rows in the canopy an additional replicate double row is shown in A,C for which the same values were used as in the other row.


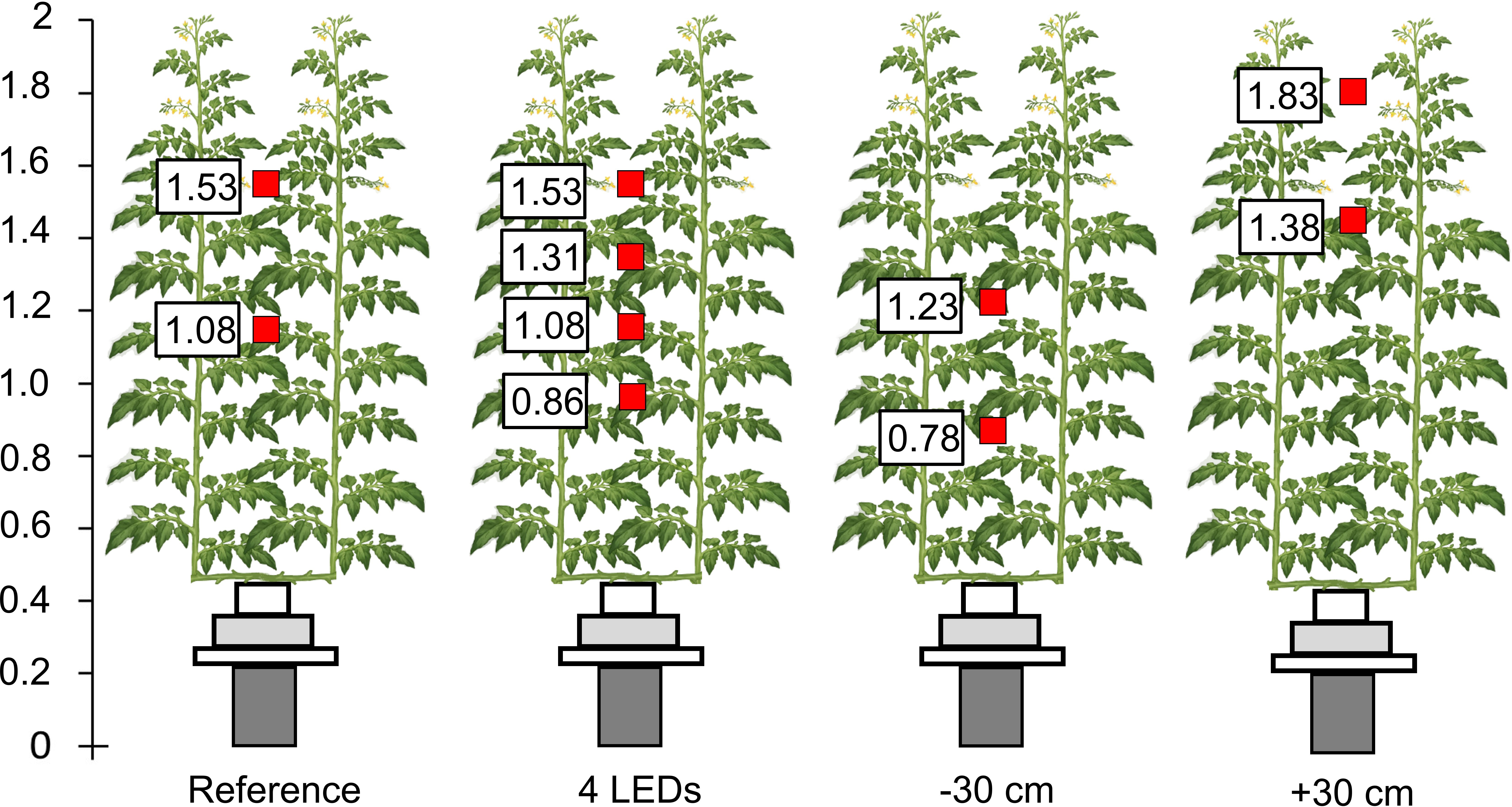


Figure S4: Relationship between measured and modelled incident PPFD (as observed by upward-facing sensors at different heights) in a greenhouse with a tomato crop illuminated by top lighting for model scenarios (A) reference simulation; (B) leaf length +25%; (C) stem width +50% (D); apex height +30 cm. The different symbols represent different distances to the middle of the row canopy, whereas the symbol color represents height from the floor. Reference apex height was 200 cm.


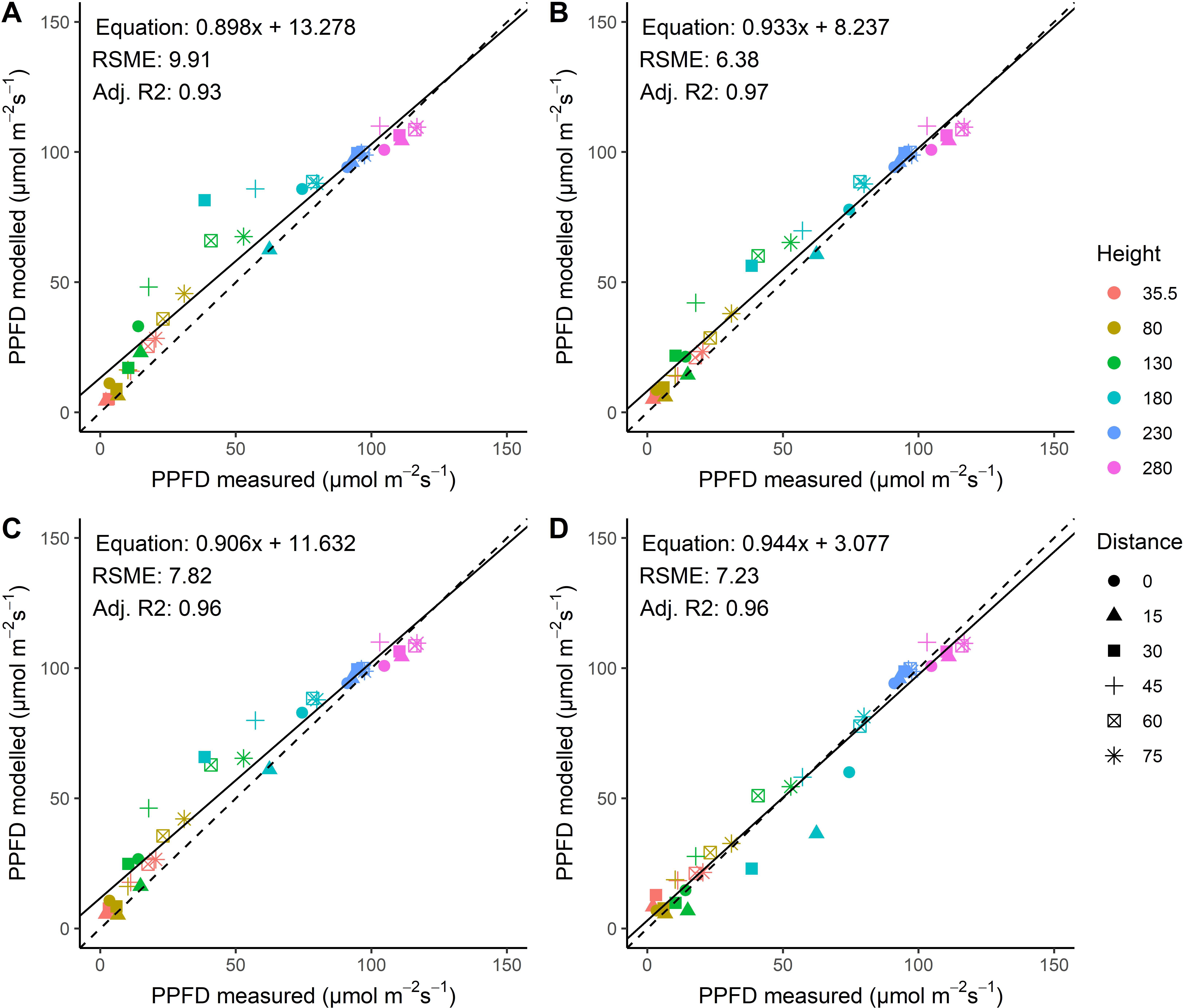


Figure S5: Relationship between measured and modelled upward-facing line sensor incident PPFD values in a greenhouse with a tomato crop illuminated by top lighting for model scenarios (A) reference simulation;

(B) leaf length +25%; (C) stem width +50% (D); apex height +30 cm. The data point shape is representing the distance to the middle of the row canopy, whereas the data point color represents height from the floor. Reference apex height was 200 cm.


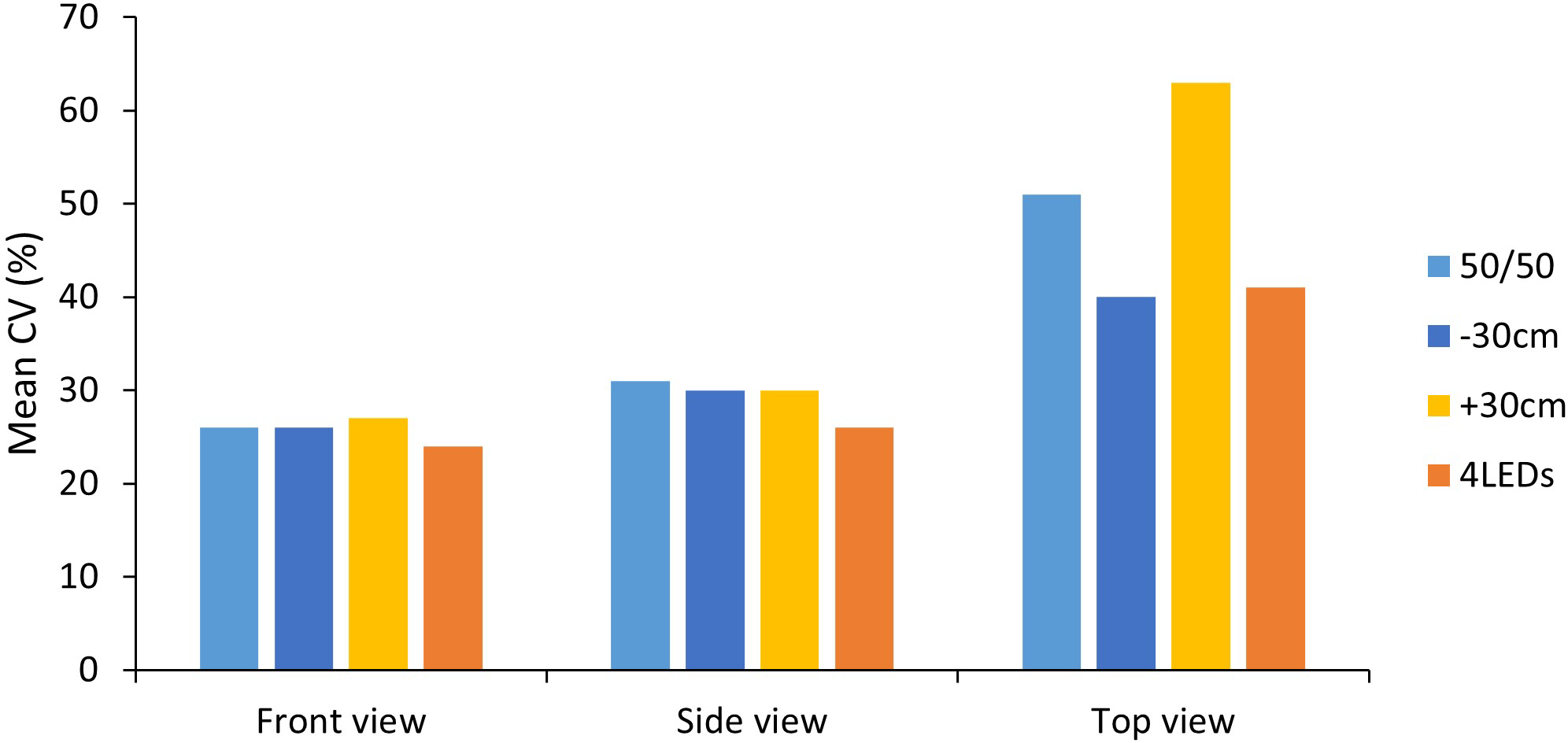


Figure S6: Sensitivity analysis of the relationship between mean coefficient of variation (CV) within voxels of absorbed canopy PPFD with differing simulation scenarios, evaluated for the front, side and top view of the canopy. Number and position of LED modules as well as standard deviation of architecture are evaluated. Simulation scenarios are performed with 50% intra-canopy lighting and the sensitivity analysis scenarios are follows; ’50/50’ is the reference simulation; ’-30 cm’ has the intra-canopy lighting LED modules lowered from 108 and 153 cm to 78 and 123 cm; ’+30 cm’ has the intra-canopy lighting LED modules raised from 108 and 153 cm to 138 and 183 cm; ’4 LEDs’ has two additional intra-canopy lighting module locations on 81 and 131 cm. Each voxel had the dimensions of 7.5 cm width and length and a depth reaching 6 plants in each row (Front View), 8 plants across four double rows (Side View) or whole plant height (Top View).
